# How should we assess knowledge translation in research organizations; designing a knowledge translation self-assessment tool for research institutes (SATORI)

**DOI:** 10.1186/1478-4505-9-10

**Published:** 2011-02-22

**Authors:** Jaleh Gholami, Reza Majdzadeh, Saharnaz Nedjat, Sima Nedjat, Katayoun Maleki, Mahnaz Ashoorkhani, Bahareh Yazdizadeh

**Affiliations:** 1 School of Public Health, Tehran University of Medical Sciences, Tehran, Iran; 2 Knowledge Utilization Research Centre (KURC), Tehran University of Medical Sciences, Tehran, Iran

## Abstract

**Background:**

The knowledge translation self-assessment tool for research institutes (SATORI) was designed to assess the status of knowledge translation in research institutes. The objective was, to identify the weaknesses and strengths of knowledge translation in research centres and faculties associated with Tehran University of Medical Sciences (TUMS).

**Methods:**

The tool, consisting of 50 statements in four main domains, was used in 20 TUMS-affiliated research centres and departments after its reliability was established. It was completed in a group discussion by the members of the research council, researchers and research users' representatives from each centre and/or department.

**Results:**

The mean score obtained in the four domains of 'The question of research', 'Knowledge production', 'Knowledge transfer' and 'Promoting the use of evidence' were 2.26, 2.92, 2 and 1.89 (out of 5) respectively.

Nine out of 12 interventional priorities with the lowest quartile score were related to knowledge transfer resources and strategies, whereas eight of them were in the highest quartile and related to 'The question of research' and 'Knowledge production'.

**Conclusions:**

The self-assessment tool identifies the gaps in capacity and infrastructure of knowledge translation support within research organizations. Assessment of research institutes using SATORI pointed out that strengthening knowledge translation through provision of financial support for knowledge translation activities, creating supportive and facilitating infrastructures, and facilitating interactions between researchers and target audiences to exchange questions and research findings are among the priorities of research centres and/or departments.

## Background

Knowledge translation, considered as both strategies and processes, has received worldwide attention in the application of health research for decision making in many universities in recent years [1,2]. Some universities and organizations see the process as a priority [3]. The necessity for the optimal utilization of research has become even more prominent in low- and middle-income countries which suffer resource shortage [4]. Knowledge translation, on the other hand, is a complex, non-linear, ongoing and dynamic process which cannot be achieved easily [4].

Knowledge translation theories have developed considerably in recent decades, and various models have been put forward to explain them [5,6]. Even though some think there is basically no need for such theories [5,7] many researchers believe a model-based framework, adopted at different levels ranging from individual to group to organizational behaviour, is required to design and implement effective and assessable interventions for turning research results into decision making [8,9].[5]. Various knowledge translation models have been designed in different fields of study (i.e., social sciences, nursing, and health services) and for a variety of organizations, each one of which emphasizes one aspect of knowledge translation. However, there is no extensive model which can be used in all situations. Basically, such a model cannot exist, because Knowledge translation strategies must be attuned to the groups' and organizations' needs and structures [7]. Only a few models have been designed for examining the performance of research organizations (e.g., universities) [5,6,10]. Therefore, the knowledge translation model was designed at Tehran University of Medical Sciences (TUMS) as a framework to identify knowledge translation capacities and weaknesses in the university and to suggest appropriate and necessary interventions, a detailed report of which has already been published elsewhere [11].

Since one way of applying models is to design and develop appropriate tools on the basis of an available model, a tool was designed to assess knowledge translation in research institutes using the 'Knowledge Translation Model of Tehran University of Medical Sciences' [12]. Such a move can not only transform a theoretic model into applicable knowledge but also helps identify strengths and weaknesses of knowledge translation in universities, as knowledge-producing organizations, and subsequently promote the utilization of knowledge in the country.

In Iran Knowledge translation has two aspects. Firstly, the Iranian healthcare system, is an integrated structure formed following the integration of the Ministry of Health into medical education in 1985 [13]. The system has potentially prepared the ground for health knowledge translation [14]. Secondly, the production of science has followed a rapid pace in recent years, pointing out the fact that the translation of this rapidly-growing knowledge needs special attention [14][15].

Apart from designing a self-assessment tool for knowledge translation activities in research-producing institutes, TUMS research centres and faculties, this pilot study uses the tool to assess the weaknesses and strengths of knowledge translation in the university.

## Methods

### Tool Development

The TUMS model provided a framework for the development of a self-assessment tool for knowledge translation [11]. All the determinant factors of knowledge translation in the model were summarized in homogenous domains, and a preliminary draft was prepared.

To ensure internal validity, five experts were asked to give their impression on each statement by 'thinking aloud', and necessary changes were subsequently made if the items did not convey the meanings we had in mind. Then, the tool was discussed for the sequence of questions and content validity among 23 researchers from five universities around the country and final modifications were implemented on the basis of their feedback.

The finalized version of the tool consists of 50 statements in four main domains (Additional file 1 and 2). Each item covers at least one of the determinant factors of knowledge translation, needing to be evaluated.

The four main domains and their sub-domains are as follows:

1- The question of research: Do we identify decision makers' research needs and convert them into research questions? This covers two sub-domains of resources (four statements) and strategies (eight statements)

2- Knowledge production: Do we produce useful evidence for decision making? (nine statements)

3- Knowledge transfer: Do we have appropriate means for disseminating the organization's research results to their target audiences? This covers two sub-domains of resources (nine statements) and strategies (16 statements)

4- Promoting the use of evidence: Do we help decision makers utilize research results better? (four statements)

By 'resources' we mean all the financial, equipment, legal and human resources that have been provided by the organization for knowledge translation activities. And by 'strategies' we mean all the steps taken in the concerned domains.

The tool permits the self-assessment of the organization through group discussions and consensus by its members, rather than by survey method. To assess an institute, the research authorities and its selected researchers should complete the tool. It would be preferred if other stakeholders of the researches carried out in the organization also participate in the meeting. First, the participants list the organization's research target audiences. Then, they review each statement and rate each item on a Likert scale upon discussion, exchange of ideas and overall consensus.

Each statement will assess at least one of the aspects influencing knowledge translation. And each item will secure a score that would cover a range of five options, ranging from 'the situation is good and needs no intervention' to 'the situation is quite unfavourable and/or there is a dire need for intervention'.

The results of the assessment will not be considered as the overall *score *obtained by the organization regarding knowledge translation activities. Instead, they will be used to identify the strengths and weaknesses of the organization for future interventions. This is why the tool is considered as a guide and not a questionnaire for cross-sectional study purposes.

### Reliability Assessment

To study the reliability of the tool using intra class correlation, 21 researchers in three research centres answered the self-assessment tool twice, at two-week intervals.

Internal consistency with Cronbach's alpha was estimated by having 45 researchers from five research centres (24 persons in addition to the first 21 researchers) complete the tool. Items having lower reliability were then revised.

### TUMS Pilot Testing

TUMS is the oldest and largest centre for health sciences in Iran, consisting of seven faculties, over 1,250 faculty members, 47 research centres and 16 teaching hospitals. Also, it holds the greatest share of medical publications in Iran [15].

Among the 47 TUMS research centres and departments, 12 research centres and eight departments were chosen to participate. One department was chosen from each faculty; in the medical faculty, a clinical and a basic science department were selected. Selection criteria included (a) Holding regular research council meetings (b) Willingness to participate in the study.

The selected research groups and centres were invited to participate in the study, and were asked to introduce one of their research council members as a focal point as a contact person. The three-hour briefing session was held to familiarize the focal points with the tool, and as a drill they completed it on their own.

The questionnaire was thereafter completed by the members of the research council and the researchers from different centres and departments. An average of six individuals participated in these sessions. In order to assure the quality of the study, the focal points were asked to arrange the research council meeting in each centre and inform the research team of its time so they would be available to answer possible questions regarding the tool.

The research team answered the questions on phone in order to avoid any possible information bias caused by their presence in the meeting.

### Data analysis

To assess each statement and domain, the mean score of each statement was calculated. The option 'the situation is quite unfavourable and/or there is a dire need for intervention' scored 1 and 'the situation is good and needs no intervention' scored 5 points. The statements which obtained the highest and lowest score were identified. To determine the lowest and highest scores, the quartiles were used.

To observe ethical considerations, the participants were told that writing down their names or the name of their centre was not necessary. The research was approved by the Ethical Board Committee of TUMS.

## Results

### Tool reliability

In addition to the domains, the intra-class correlation coefficient (ICC) was calculated for each of the statements. The three domains of 'the question of research, knowledge production and knowledge transfer' had an ICC and Cronbach's alpha higher than 0.70, whereas the figure was lower than 0.70 for 'promoting the use of evidence' (Table 1). A low Cronbach's alpha was likely considering the small number of questions and the nature of the statements in the fourth domain. A small ICC, however, showed the low reliability of the statements in the very domain. Considering the importance of these statements, they were kept in the tool in spite of their low reliability. For better clarification of the statements, we specified them with an asterisk in the guide, and asked the focal points to give further explanations to the participants.

**Table 1:** The tool's reliability indicators

Domain	Cronbach's alpha	Intra class correlation
The research question	0.79	0.94
Knowledge production	0.70	0.87
Knowledge transfer	0.86	0.90
Promoting the use of evidence	0.27	0.48

### Assessment of research centres and departments

All the selected centres (12 research centres and eight departments) completed and delivered the self-assessment questionnaire.

The mean score of each statement was calculated for the centres and departments. The minimum and maximum mean scores obtained in various statements were 1.15 and 3.95 respectively. Tables 2, 3, 4 and 5 represent the mean scores and standard deviations of each of the statements in the four domains. The statements which gained the highest and lowest scores respectively have been specified in these tables.

**Table 2:** The mean score and standard deviation for each statement in 'question of research'

	Statement (resources)	Mean (SD) N = 20*
1	In our organization there is a comprehensive list of organizations that can use our research results.	2.00 (1.21)
2	The particulars of each unit's researchers and their capabilities are made available to other organizations through a databank.	2.15 (1.23)
3	A website and/or data bank is available in our organization for notifying the research priorities of other organizations	2.21 (1.23)
4	Compared to the organization's internal budget for research, the amount of external funding is such that researchers are encouraged to use external funding.	2.00 (1.37)

	**Statement (strategies)**	

5	Regular meetings are held for the exchange and identification of research priorities of individuals and/or research-using organizations.	2.10 (1.21)
6	Individuals and decision-maker organizations know which fields our organizations' research capacities cover.	2.50 (0.95)
7	For preparing grounds for performing related research and strengthening research utilization, our organization holds regular and purposeful meetings with decision-makers (managers and policy makers) for extending cooperation and using mutual capacities (establishment of knowledge network)	1.89 (1.10)
8	Our organizations' research priorities are determined through meetings with executive organizations' representatives and/or users of research results (like community representatives, patients etc)	1.50 (0.83) ↓
9	Our organizations' research priorities are compiled and its up-to-date list is available to the organizations' researchers.	3.15 (1.23) ↑
10	Compared to the internal process, the external grant securing process is such that researchers are encouraged to use external funding. (the extra-organizational part of the process)	1.95 (1.39)
11	In case of external funding, researchers can use these for research matters easily and in a short period of time. (the intra-organizational part of the process)	3.18 (1.33) ↑
12	Incentives exist for our researchers for securing external funding.	2.50 (1.10)

↓ The statements which obtained the lowest scores

↑ The statements which obtained the highest scores

*Each of the twenty centres and departments that participated in the study agreed upon a single score which was used in calculating the mean score.

**Table 3:** The mean score and standard deviation for each statement in 'knowledge production'

	Statement	Mean (SD) N = 20
1	Researches that result in production of 'actionable messages' with a high level of evidence (such as regular systematic reviews and/or clinical guideline development activities) are considered priorities of research and granted funds.	2.45 (1.43)
2	The groups which will use the results of research participate in its conduction and/or design.	2.15 (0.90)
3	Our impression is that the users of research results trust the quality of the researches done in the organization.	3.95 (0.83) ↑
4	Quality assurance program is required for each research (data gathering protocol and/or training the research workers)	3.20 (0.89) ↑
5	Quality control is carried out while research is being conducted (internal monitoring of the executive program by the research group and/or external supervision)	2.95 (1.10) ↑
6	The gap between 'presentation of the research proposal' and 'beginning of the research' is reasonable (the process of reviewing the research proposal)	2.85 (1.27) ↑
7	While designing the research proposal and performing the projects researchers are aware that applied projects should reach results in good time (the projects duration and absence of delay in performing them)	3.75 (0.91) ↑
8	The gap between 'end of research' and 'finalization of results in the form of a report' is reasonable (the process of presentation of research results)	3.60 (0.82) ↑
9	In research project proposals (projects whose users are service providers, managers, policy makers, patient groups and/or people) budget is considered for disseminating the results (other than being published in peer-review journals and/or attending conferences)	1.39 (0.98) ↓

↓ The statements which obtained the lowest scores

↑ The statements which obtained the highest scores

**Table 4:** The mean score and standard deviation for each statement in 'knowledge transfer'

	Statement (resources)	Mean (SD) N = 20
1	Researchers are familiar with the topic of knowledge translation and how to perform it.	2.63 (1.21) ↑
2	Our researchers have communication skills for knowledge transfer.	2.95 (1.36)↑
3	Our researchers can use the services of those familiar with knowledge transfer skills (the presence of individuals in our organization who work with this objective; and/or make contracts with individuals and institutions outside our organization)	2.00 (1.25)
4	Our researchers have the necessary financial resources for preparing content appropriate to the target audience.	1.39 (0.92)↓
5	Our researchers have the necessary equipment for preparing content appropriate to the target audience.	2.16 (1.21)
6	The necessary structure (like office and/or organizational unit) and/or manpower is available for strengthening knowledge transfer in our organization, considering the produced amount of research-based knowledge transferable to the decision makers	1.45 (0.76)↓
7	The framework of research projects' final reports is such that decision makers can easily point out the actionable message.	2.50 (1.05)
8	Intellectual property rights exist which support researchers who help disseminate research results prior to their publication in journals.	1.15 (0.37)↓
9	There are criteria for evaluation of researchers' knowledge transfer activities in our organization.	1.55 (0.83) ↓

	**Statement (strategies)**	

10	In our organization there is a process that determines which research results can be transferred (keeping in mind the fact that not every research result is transferable) to the target audiences (apart from other researchers and funders)	1.42 (0.69)↓
11	In our organization, all research results are peer reviewed prior to knowledge dissemination or transfer.	3.70 (1.17)↑
12	Our researchers convert their research results into actionable messages appropriate to the target audience.	2.35 (1.35)
13	Our researchers have adequate time for preparing content appropriate to the target audience.	2.16 (0.90)
14	Our researchers have the necessary incentives for performing knowledge transfer (rewards, appropriate promotion rules)	2.42 (1.17)
15	Knowledge transfer and utilization of research results exist in the general program of research methodology training	1.67 (0.84)
16	A list of all the (research result users) is prepared for each research project.	2.35 (1.14)
17	Our organizations' research managers are aware of the researchers needs (separately for each study field-group etc) in the field of knowledge transfer, and perform proper interventions for them.	1.50 (0.71)↓
18	The format of peer review journals which publish research results is such that the decision makers are easily informed of the actionable message when necessary.	2.45 (1.19)
19	The gap between sending the article and its publication in journals is such that the interventions that result from research can be implemented in reasonable time (considering the need for prompt availability of research results to decision makers).	2.35 (1.18)
20	Researchers can provide the results of their research through the web and/or electronic banks.	1.95 (1.23)
21	Meetings are held for presentation of research results to decision makers.	1.47 (1.02)↓
22	Our organization has regular communications with public and private media and target audiences (like publications related to women and youth) for transfer of research-based evidence.	1.55 (1.00)↓
23	Evidence-based decision making (based on domestic and/or foreign research) is among the subjects of research in our organization.	1.80 (1.24)
24	Our researchers study the extent to which decision makers utilize our organizations' research results.	1.55 (0.89)↓
25	Our researchers identify the potential barriers of behavioral change in decision makers for utilizing their research results.	1.60 (0.94)

↓ The statements which obtained the lowest scores

↑ The statements which obtained the highest scores

**Table 5:** The mean score and standard deviation for each statement in 'promoting the use of evidence'

	Statement	Mean (SD) N = 20
1	We conduct education programs such as 'evidence-based medicine' or 'evidence-based decision making' for service providers and/or managers	1.80 (1.15)
2	Systematic reviews and clinical guidelines...etc that strengthen evidence-based decision making are produced in our organization.	1.95 (1.28)
3	Our researchers play an active role in technical committees that help in decision making (executive organizations' decision making, hospital management and also groups supporting the health of patients and people)	2.53 (1.39)↑
4	We send decision makers reminders to follow the research results that we've previously sent them	1.30 (0.73) ↓

↓ The statements which obtained the lowest scores

↑ The statements which obtained the highest scores

The statements which obtained the lowest scores represented issues that were believed to be in a poorer condition and needed interventions. The contrary was true for the statements which obtained the highest scores. The items with a mean score lower than 1.6 (the first quartile) included setting research priorities with research users, financial resources, infrastructures, researchers' intellectual rights, researchers' need assessment and evaluation of their knowledge translation activities, direct communication with media and research users and follow-up of research utilization.

The following statements reflect weaknesses in the university due to their low scores (less than 1^st ^quartile): determining research priorities through meetings with stakeholders (statement 8 from 1^st ^domain), considering budgets in proposals and securing funds for knowledge translation (statement 9 from 2^nd ^domain and statement 4 from 3^rd ^domain), communication between researchers and research target audiences and follow-up of utilization of research results (statements 21, 22 and 24 from 3^rd ^domain and statement 4 from 4^th ^domain), infrastructure (statement 6 from 3^rd ^domain) and supportive regulations and measures for knowledge translation activities (statements 8, 9,10 and 17 from 3^rd ^domain).

On the other hand, dissemination of research priorities (statement 9 from 1^st ^domain), facilitating the receipt of grants from other organizations (statement 11 from 1^st ^domain), quality of research (statements 3, 4 and 5 from 3^rd ^domain), timeliness of granting, conducting and providing research results (statements 6,7 and 8 from 1^st ^domain) and being acquainted with knowledge translation and having communication skills (statements 1 and 2 from 3^rd ^domain) were in the 4^th ^quartile of the scores and reflect strengths of the university.

Statements which obtained the lowest scores pertained to knowledge transfer strategies (5 statements), knowledge transfer resources (4 statements), strategies for developing research questions (1 statement), knowledge production (1 statement) and promoting the use of evidence (1 statement). On the other hand, statements which obtained the highest scores (greater than the third quartile) respectively, were, from knowledge production (6 statements) strategies for developing research questions (2 statements), knowledge transfer resources (2 statements) and strategies (1 statement), and promoting the use of evidence (1 statement).

The mean domain score was also calculated. Knowledge production obtained the highest score with a mean score of 2.92 ± 0.83, whereas promoting the use of evidence obtained the lowest (1.89 ± 0.55). The mean score of the research question domain was 2.26 ± 0.50, and the knowledge transfer domain obtained a mean score as low as 2.00 ± 0.59.

## Discussion

The SATORI tool (SATORI: a Japanese Buddhist term for enlightenment, literally meaning 'understanding') provides a way to operationalize the TUMS knowledge translation model.

This tool consists of 50 statements about requisites, resources and strategies for facilitating knowledge translation in research institutes. Use of the tool enables research managers and researchers to identify strengths and weaknesses of knowledge translation within their institution and to subsequently develop interventions that could improve their organization's KT infrastructure and capacity.

This tool was developed to assess knowledge translation activities from the "push side" perspective, meaning activities which are undertaken by researchers or research organizations to transfer research results to target audiences.

Research managers and researchers can identify the strengths and weaknesses of their organization regarding knowledge translation upon using this 50-statement tool, and work toward identifying solutions for the improvement of the organization's infrastructure and capacity. Actually, this tool is a complement to the "Is research working for you?" which is a self-assessment tool and discussion guide designed by the Canadian Health System Research Foundation (CHSRF) to examine "pull side" activities; activities performed by health services management and policy organizations to benefit from research evidences [16-19].

In many countries, evaluation of research outputs in academic units is used as a method of allocating funds, it is also used as a management tool to monitor the performed activities [20]. Nonetheless, methodologies for assessing the KT capacity of research organizations are still in their infancy and until the execution of this study the authors had not come across a tool that could evaluate the capacities of research organizations (university, faculty, public and private research centres and groups), nor the obstacles faced in knowledge translation. To our knowledge, existing questionnaires for assessing knowledge translation activities are completed by health researchers individually [21,22]. A framework has, however, been proposed by Lavis to evaluate 'linking research to action' measures from 'Push, Pull, and Exchange' aspects at national levels [19]. A questionnaire has also been designed by Tugwell et al to assess the capacity of low and middle-income countries for performing equity-oriented research at national levels [23]. Other studies conducted in this regard have mostly qualitatively assessed the quality of knowledge translation at organizational levels [4].

The comparison of organizational strengths and weaknesses, based on the scores gained through this questionnaire can help officials define intervention priorities. Prioritization, however, could not be done solely by comparing the statements' raw scores, since all these statements have equal weights but different significance and generalization values. For example, sending a reminder regarding the research results to decision makers is a simple process in need of few resources. Although the establishment of an organizational unit for improving knowledge translation is a costly strategy; it may be beneficial for developing strategies to promote KT, such as designing a guideline for publishing the results, and the development of regulations to support the intellectual rights of the researchers. The following steps can help prioritize KTE interventions: (1) preparing a list of the statements which have obtained low scores on the self-assessment test, (2) formulating intervention options, and (3) assessing the organizational context and different aspects such as feasibility, cost and chance of success for each intervention.

The pilot study conducted across 20 TUMS centres and departments showed that while conducting research and producing knowledge are performed with appropriate quality and timeliness in the university, there are significant weaknesses in the interactions between researchers and their research target audiences (both in selecting research priorities *and *transferring the results), securing the financial resources and following supportive regulations for knowledge translation activities. The comparison of the mean scores obtained in different domains confirmed that research capacity and knowledge production is acceptable but there are certain weaknesses in the aptitude of the last two domains of knowledge transfer and promoting the use of evidence.

In view of the weaknesses identified in our study, the main interventions needed for TUMS include:

1) Facilitating knowledge translation activities through provision of financial resources required for these activities;

2) Facilitating knowledge translation activities through creating supportive and facilitating infrastructures for these activities;

3) Facilitating interactions between researchers and target audiences to exchange questions and research findings.

The challenges faced by TUMS in knowledge translation issues are somewhat similar to those faced by other countries [24-26]. Though many investors in developed countries financially support knowledge translation activities [27], and have established certain structures and regulations to strengthen knowledge translation, the interaction between researchers and target audiences is still a major concern. Most of the interventions proposed to strengthen knowledge translation, even in developed countries, focus on facilitating and enhancing the interaction between researchers and research users [28-30].

The aim of this study was to develop a tool to identify the obstacles (weaknesses) in knowledge translation in TUMS-affiliated faculties and research centres. It seems the knowledge translation-related issues are somewhat similar in research organizations located in developed and developing countries. As a result, the standardized-version of the tool can be used in other research organizations.

The SATORI has some particular aspects. First of all, this tool has been prepared on the basis of the "Knowledge Translation Model at Tehran University of Medical Sciences" on the grounds of domestic studies and a comprehensive literature review on knowledge translation barriers. And in addition to being used in Iran's academic environment, we believe it could be applied to other countries' research organization settings as well. This tool covers the most important activities, necessary resources and facilitating strategies for knowledge translation at the organizational level, and similar to its Canadian counterpart, this tool can also be used for re-evaluation of the organization's promotion in knowledge translation [17]. In addition, discussion and dialogue is possible concerning each statement of the tool that could lead to identification of intervention(s) regarding weaknesses of the organization. Finally, different perspectives could be elaborated upon and considered during the prioritization of interventions, since the discussion group consists of a variety of different stakeholders including members of research councils, research managers, researchers and research users.

## Conclusions

In addition to identifying the weaknesses in the KT capacities of research centres and organizations, the tool can help develop interventional priorities to solve these barriers and difficulties. It seems that strengthening knowledge translation in TUMS will take place through the provision of financial support for knowledge translation activities, creating supportive and facilitating infrastructures, and facilitating interactions between researchers and target audiences.

## Competing interests

The authors declare that they have no competing interests.

## Authors' contributions

RM proposed the idea and designed it. He also participated in the tool design, statistical analysis and preparing the manuscript. JG participated in the tool design, data collection management, statistical analysis, initial draft preparation and subsequent manuscript corrections. SN participated in the study design, tool design and manuscript correction.

KM translated the manuscript and assisted in interpreting the statistical analysis and manuscript correction. MA assisted in the tool design, data collection and manuscript correction. BY contributed to the development of the tool as well as the development of the report. All authors approved the final manuscript.

## Supplementary Material

Additional file 1 **Knowledge Translation Self Assessment Tool for Research Institutes (SATORI) in English**. Click here for file

Additional file 2 **Knowledge Translation Self Assessment Tool for Research Institutes (SATORI) in Farsi**. Click here for file
