# Supplementary material for: How should we assess knowledge translation in research organizations; designing a knowledge translation self-assessment tool for research institutes (SATORI)
Source: Health Res Policy Syst. 2011 Feb 22;9:10. doi: 10.1186/1478-4505-9-10 (PMC3053266; doi:10.1186/1478-4505-9-10)
Supplement: Additional file 1 — Knowledge Translation Self Assessment Tool for Research Institutes (SATORI) in English. [file 1478-4505-9-10-S1.PDF]

Tehran University of Medical Sciences

Knowledge Translation
Self Assessment Tool for Research Institutes (SATORI)

Does our research center (or school) take possible measures for knowledge translation?

Knowledge Utilization Research Center (KURC)

What is this tool?

SATORI meaning “understanding” is a Japanese Buddhist term for enlightenment. The tool was developed through a research conducted in Tehran University of Medical Sciences. In this qualitative study the barriers to research-based knowledge translation in Tehran University of Medical Sciences and also the obstacles mentioned in studies performed elsewhere have been identified through various literature reviews. The result of this research project was to design a Knowledge Translation Model in Tehran University of Medical Sciences.

Majdzadeh R, Sadighi J, Nedjat S, Shahidzade A, Gholami J. Design of a Knowledge Translation Model in Tehran University of Medical Sciences for Research Utilization. Journal of Continuing Education in the Health Professions. 2008; 28(4): 270

The application of this model led to the development of a self-assessment tool with which research authorities and researchers of any institute (is it the university, school, private or public research center) can identify the barriers to knowledge transfer in their own organization and suggest appropriate solutions to improve the situation.
The *Canadian Health System Research Foundation* has prepared a similar tool which addresses executive organizations which are research users (unlike this tool which addresses knowledge-producing organizations). The designers of this tool emphasize that the content of the tool can be changed according to the organizations’ specific characteristics. It should be noted that the validity and reliability of this tool has been approved and its potency has been tested in pre-tests conducted in some universities and research centers.
Please send your suggestions to rezamajd@tums.ac.ir and or kurc@tums.ac.ir in order to receive future versions of this tool.

How to use this tool?

Invite your research center/schools researchers and research council members. It is better to invite the representatives of your organization’s research users as well. Distribute the tool among them before the meeting, allowing them to go through the *items* and add some if they want to.
In many parts of this text research users and decision makers have been mentioned. Please note that these groups may be policy makers, executive managers, hospital managers, health service providers such as physicians, midwives, nurses, pharmacists, dentists and … and also patient groups or people.
Please ask your colleagues to **list the users of their research results.** Then, review the *items* in each of the four sections. Discuss each item and give a score to them. Some of the items have been marked with a (*) sign. These are questions which had an Intraclass Correlation Coefficient (ICC) of less than 0.7 in the reliability assessment. You are therefore requested to provide the necessary explanations. It is worth mentioning that available studies and

literature review have shown that these statements cover important points in each organization, and so their presence in this tool is of significance.
The obtained results outline the problems your organization faces with respect to knowledge transfer. It is better to choose the more important problems at the beginning. This is why you should **‘prioritize’**. Then suggest **‘interventions’** for those with higher priority. You should:

1- Consider **your research users,**
2- **Score the *items*** of self-assessment in this tool,
3- **Prioritize** the problems identified,
4- Choose **interventions** for the problems that are priority.

This tool has been designed in four sections:
1-*The question of research*: Do we identify decision makers’ research needs and convert them into research questions?
2-*Knowledge production*: Do we produce evidence that is useful for decision making?
3-Knowledge transfer: Do we have appropriate means for disseminating the organizations’ research results to their target audiences?
4-*Promoting the use of evidence:* Do we help decision makers utilize research results better?
It should be kept in mind that many of the topics that strengthen the research may also reinforce knowledge transfer. Some of the statements refer to common topics on strengthening research, including priority setting (the question of research) and producing evidence (knowledge production). However the third part that refers to *knowledge transfer* is more specific and focused. The other point is that this is not all that can be done for strengthening knowledge translation; the main point is to introduce interventions for ‘research-based knowledge translation’ in research institutes. We should remember that alongside this self-assessment tool is another tool for decision making organizations.

In the beginning we will define a few terms:

- **‘knowledge translation’**

“Knowledge translation is the exchange, synthesis and ethically-sound application of knowledge—within a complex system of interactions among researchers and users—to accelerate the capture of the benefits of research through improved health, more effective services and products, and a strengthened health care system.”

- **Collaboration**

Three models can be described with respect to decision makers (policy makers) collaboration:

1. **Formal Supporter**: In this process the decision makers are not actively involved in the research process; they are unaware of the ongoing research activities; they support the goals of research; they legalize the execution of research and facilitate access to the resources.

2. **Responsive Audience**: In this condition the decision makers are somehow active and respond to the researchers' ideas, provide the information they need, give the necessary counseling and set up necessary connections. Connections are initially made by the researchers through consultations and meetings in which ideas are exchanged. The researcher informs the decision makers of the research activities through written reports.
3. **Integral Partner**: In this condition the decision makers are completely involved in the research process and are influential members of the team. The connections are initially made by the decision makers through meetings and exchange of ideas with the researchers. Research reports are compiled after coordination and collaboration among the researchers and policy makers. The decision makers are influential in the formation of the research process and its results.

Research producing organizations

By research producing organizations we mean any institution that has the ability to define a research project and approve it (financial approval is not necessarily in mind, any institution that has a research council and can scientifically approve projects is), such as research centers and schools.

Please keep the following points in mind while answering the questions:

- The items cover a range of answers, please choose the most appropriate option
- If any explanation is necessary please write it down in the 'explanations' section
- If a question does not comply with your organization write it down in the 'explanations' section

Section 1: The question of research

Do we identify decision makers' research needs and convert them into research questions?

		The situation is quite unfavorable and/or there is a dire need for intervention	2	3	4	The situation is good and needs no intervention
1-1	In our organization there is a comprehensive **list** of **organizations** that can use our research results.<br>Explanations:<br>…………………………………………...........	1	2	3	4	5
1-2	The **particulars** of each unit's **researchers** and their capabilities are made available to other organizations through a **databank**.<br>Explanations:<br>……………………………………………….	1	2	3	4	5
1-3	**Regular meetings** are held for the exchange and identification of **research priorities** of individuals and/or research-using organizations[1].<br>Explanations:<br>……………………………………………….	1	2	3	4	5
1-4	Individuals and decision-maker organizations know which fields **our organizations' research capacities** cover.<br>Explanations:<br>……………………………………………….	1	2	3	4	5
1-5	For preparing grounds for performing relevant research and strengthening research utilization, our organization holds **regular** and **purposeful meetings** with decision-makers (managers and policy makers) for **extending cooperation** and using **mutual capacities** (establishment of a knowledge network).<br>Explanations:<br>……………………………………………….	1	2	3	4	5

[1] Please note that here the research priorities of research using organizations is in mind

		The situation is quite unfavorable and/or there is a dire need for intervention	2	3	4	The situation is good and needs no intervention
1-6	A website and/or data bank is available in our organization for notifying the **research priorities of other organizations.**<br>Explanations:<br>…………………………………………….	1	2	3	4	5
1-7	**Our organizations' research priorities** are determined through meetings with executive organizations' representatives and/or **users of research results** (like community representatives, patients etc)[1].<br>Explanations:<br>…………………………………………….	1	2	3	4	5
1-8	**Our organizations' research priorities** are compiled and its **up-to-date list** is available to the organizations' researchers[2].<br>Explanations:<br>…………………………………………….	1	2	3	4	5
1-9	Compared to the organization's internal budget for research, the amount of external funding is such that researchers **are encouraged to use external funding.**<br>Explanations:<br>…………………………………………….	1	2	3	4	5
1-10	Compared to the internal process, the external grant securing process is such that researchers are **encouraged to use external funding.** (the extra-organizational part of the process).<br>Explanations:<br>…………………………………………….	1	2	3	4	5
1-11	In case of **external funding**, researchers can use these for research matters **easily and in a short period of time**. (the intra-organizational part of the process).<br>Explanations:<br>…………………………………………….	1	2	3	4	5

[1] Please note that here the research priorities of organizations doing research is in mind.
The research producing organizations priorities may be based on researchers' ideas, futuristic views, and evaluation of community or other organizations' needs. However, they may differ from other organizations research needs (which may be one or few of the reasons mentioned).

		The situation is quite unfavorable and/or there is a dire need for intervention	2	3	4	The situation is good and needs no intervention
1-12	**Our researchers** have the **incentives for securing external funding.**<br>Explanations:<br>………………………………………………..	1	2	3	4	5
(In case you would like to add other options suitable to your organization you may add these below.)						
		1	2	3	4	5
		1	2	3	4	5
		1	2	3	4	5
		1	2	3	4	5

Section 2: Knowledge production

Do we produce useful evidence for decision making?

		The situation is quite unfavorable and/or there is a dire need for intervention	2	3	4	The situation is good and needs no intervention
2-1	Research studies that result in **production** of **'actionable messages'** with a high level of evidence (such as regular systematic reviews and/or clinical guideline development activities) are considered priorities of research and granted funds.<br>Explanations:<br>…………………………………………….	1	2	3	4	5
2-2	The groups which will use the results of research **participate** in its conduction and/or design.<br>Explanations:<br>…………………………………………….	1	2	3	4	5
2-3	Our impression is that the **users** of research results trust the **quality** of the research done in the organization.<br>Explanations:<br>…………………………………………….	1	2	3	4	5
2-4	**Quality assurance** program is required for each research (data gathering protocol and/or training the research workers).<br>Explanations:<br>…………………………………………….	1	2	3	4	5
2-5	**Quality control** is carried out while research is being conducted (internal monitoring of the executive program by the research group and/or external supervision).<br>Explanations:<br>…………………………………………….	1	2	3	4	5
2-6	The gap between 'presentation of the research proposal' and 'beginning of the research' is reasonable (the process of **reviewing the research proposal**).<br>Explanations:<br>…………………………………………….	1	2	3	4	5

		The situation is quite unfavorable and/or there is a dire need for intervention	2	3	4	The situation is good and needs no intervention
2-7	While designing the research proposal and performing the projects researchers are aware that applied projects should reach results in good time (**the projects duration** and **absence of delay in performing them**)<br>Explanations:<br>………………………………………………..	1	2	3	4	5
2-8	The gap between 'end of research' and 'finalization of results in the form of a report' is reasonable (**the process of presentation of research results**).<br>Explanations:<br>………………………………………………..	1	2	3	4	5
2-9	In **research project proposals** (projects whose users are service providers, managers, policy makers, patient groups and/or people) **budget is considered for disseminating the results** (other than being published in peer-review journals and/or attending conferences).<br>Explanations:<br>………………………………………………..	1	2	3	4	5
(In case you would like to add other options suitable to your organization you may add these below.)						
		1	2	3	4	5
		1	2	3	4	5
		1	2	3	4	5

Section 3: Knowledge transfer

Do we have appropriate means for disseminating the organizations’ research results to their target audiences?

		The situation is quite unfavorable and/or there is a dire need for intervention	2	3	4	The situation is good and needs no intervention
3-1	In our organization there is a process that determines **which research** results can be transferred (keeping in mind the fact that not every research result is transferable) to the target audiences (apart from transferring to other researchers and funders).<br>Explanations:<br>……………………………………………….	1	2	3	4	5
3-2	In our organization, all research results are **peer reviewed** prior to knowledge dissemination or transfer.<br>Explanations:<br>……………………………………………….	1	2	3	4	5
3-3	Researchers are **familiar** with the topic of **knowledge translation** and how to perform it.<br>Explanations:<br>……………………………………………….	1	2	3	4	5
3-4	Our researchers convert their research results into **actionable messages** appropriate to the target audience.<br>Explanations:<br>……………………………………………….	1	2	3	4	5
3-5	Our researchers have **communication skills** for knowledge transfer.<br>Explanations:<br>……………………………………………….	1	2	3	4	5
3-6	Our researchers can use the **services** of those familiar with **knowledge transfer skills** (the presence of individuals in our organization who work with this objective; and/or make contracts with individuals and institutions outside our organization).<br>Explanations:<br>……………………………………………….	1	2	3	4	5

		The situation is quite unfavorable and/or there is a dire need for intervention	2	3	4	The situation is good and needs no intervention
3-7	Our researchers have the necessary **financial resources** for preparing content appropriate to the target audience.<br>Explanations:<br>……………………………………………….	1	2	3	4	5
3-8	Our researchers have the necessary **equipment** for preparing content appropriate to the target audience.<br>Explanations:<br>……………………………………………….	1	2	3	4	5
3-9	Our researchers have adequate **time** for preparing content appropriate to the target audience.<br>Explanations:<br>……………………………………………….	1	2	3	4	5
3-10	**Our researchers** have **the necessary incentives** for performing knowledge transfer (rewards, appropriate promotion rules).<br>Explanations:<br>……………………………………………….	1	2	3	4	5
3-11	Knowledge transfer and utilization of research results exist in the **general program of research methodology training**.<br>Explanations:<br>……………………………………………….	1	2	3	4	5
3-12	**A list of all the (**research result **users)** is prepared for each research project.<br>Explanations:<br>……………………………………………….	1	2	3	4	5
3-13	**The necessary structure** (like office and/or organizational unit) and/or **manpower** is available for strengthening knowledge transfer in our organization, considering the produced amount of research-based knowledge transferable to the decision makers.<br>Explanations:<br>……………………………………………….	1	2	3	4	5
3-14	Our organizations' research managers are aware of the researchers **needs** (separately for each study field-group etc) in the field of knowledge transfer, and perform proper interventions for them.<br>Explanations:<br>……………………………………………….	1	2	3	4	5

		The situation is quite unfavorable and/or there is a dire need for intervention	2	3	4	The situation is good and needs no intervention
3-15	The format of **peer review journals** which publish research results is such that the decision makers are easily informed of the **actionable message** when necessary.<br>Explanations:<br>……………………………………………….	1	2	3	4	5
3-16	**The gap between sending the article and its publication** in journals is such that the interventions that result from research can be implemented in reasonable time (considering the need for prompt availability of research results to decision makers)[1].<br>Explanations:<br>……………………………………………….	1	2	3	4	5
3-17	The **framework** of research projects' **final reports** are such that decision makers can easily point out the actionable message.<br>Explanations:<br>……………………………………………….	1	2	3	4	5
3-18	Researchers can provide the results of their research through the **web** and/or **electronic banks**.<br>Explanations:<br>……………………………………………….	1	2	3	4	5
3-19	**Meetings** are held for **presentation of research results** to decision makers.<br>Explanations:<br>……………………………………………….	1	2	3	4	5
3-20	Our organization has **regular communications with public and private media** and target audiences (like publications related to women and youth) for transfer of research-based evidence.<br>Explanations:<br>……………………………………………….	1	2	3	4	5

[1] The authors are aware that the journals that usually publish articles may be outside the organization, but it may be possible to introduce appropriate interventions in this field; for example the decision to publish a journal, or to encourage and support publication in electronic journals. This is why these questions are left open for discussion.

		The situation is quite unfavorable and/or there is a dire need for intervention	2	3	4	The situation is good and needs no intervention
3-21	**Intellectual property rights** exist which support researchers who help disseminate research results prior to their publication in journals.<br>Explanations:<br>……………………………………………….	1	2	3	4	5
3-22	**Evidence-based decision mak**ing (based on domestic and/or foreign research) is among the subjects of research in our organization.<br>Explanations:<br>……………………………………………….	1	2	3	4	5
3-23	*Our researchers study the extent to which **decision makers utilize** our organizations' **research results**.<br>Explanations:<br>……………………………………………….	1	2	3	4	5
3-24	Our researchers identify the potential **barriers of behavioral change in decision makers** for utilizing their research results.<br>Explanations:<br>……………………………………………….	1	2	3	4	5
3-25	**There are criteria for evaluation** of researchers' knowledge transfer activities in our organization.<br>Explanations:<br>……………………………………………….	1	2	3	4	5
(In case you would like to add other options suitable to your organization you may add these below.)						
		1	2	3	4	5
		1	2	3	4	5
		1	2	3	4	5
		1	2	3	4	5

Section 4: Promoting the use of evidence

Do we help decision makers utilize research results better?

		The situation is quite unfavorable and/or there is a dire need for intervention	2	3	4	The situation is good and needs no intervention
4-1	We conduct education programs such as **'evidence-based medicine'** or **'evidence-based decision making'** for service providers and/or managers.<br>Explanations:<br>…………………………………………….	1	2	3	4	5
4-2	Systematic reviews and clinical guidelines…etc that strengthen evidence-based decision making are produced in our organization.<br>Explanations:<br>…………………………………………….	1	2	3	4	5
4-3	Our researchers play an active role in technical committees that help in decision making (executive organizations' decision making, hospital management and also groups supporting the health of patients and people).<br>Explanations:<br>…………………………………………….	1	2	3	4	5
4-4	We send decision makers **reminders** to follow the research results that we've previously sent them.<br>Explanations:<br>…………………………………………….	1	2	3	4	5
(In case you would like to add other options suitable to your organization you may add these below.)						
		1	2	3	4	5
		1	2	3	4	5
		1	2	3	4	5
		1	2	3	4	5

#12, Nosrat St., 16th Azar St., Keshavarz Blvd, Tehran, Iran.
Phone/Fax: +98-21-66495859,+ 98-21-66419763,+ 98-21-66952530,
E-mail: kurc@tums.ac.ir
