# Supplementary material for: How should we assess knowledge translation in research organizations; designing a knowledge translation self-assessment tool for research institutes (SATORI)
Source: Health Res Policy Syst. 2011 Feb 22;9:10. doi: 10.1186/1478-4505-9-10 (PMC3053266; doi:10.1186/1478-4505-9-10)
Supplement: Additional file 2 — Knowledge Translation Self Assessment Tool for Research Institutes (SATORI) in Farsi. [file 1478-4505-9-10-S2.PDF]

دانشگاه علوم پزشکی و خدمات بهداشتی درمانی تهران

ابزار خودارزیابی ترجمه دانش ویژه سازمان های پژوهشی

Knowledge Translation

Self Assessment Tool for Research Institutes (SATORI)

آیا مرکز تحقیقات ما، اقدامات ممکن برای ترجمه دانش را انجام می دهد؟

مرکز تحقیقات بهره برداری از دانش سلامت

Knowledge Utilization Research Center (KURC)

این ابزار چیست؟

این ابزار حاصل یک طرح پژوهشی است که در دانشگاه علوم پزشکی تهران به انجام رسیده و طی آن تلاش شده تا موانع موجود در ترجمه دانش حاصل از پژوهش در دانشگاه علوم پزشکی تهران و همچنین موانع مطرح شده در سایر مطالعات بر اساس مرور منابع استخراج و شناسایی گردد. ماحصل این طرح پژوهشی طراحی مدل *"مدل ترجمه دانش"* در *دانشگاه بود که* در مقاله زیر ارائه شده است.

Majdzadeh R, Sadighi J, Nedjat S, Shahidzade A, Gholami J. Design of a Knowledge Translation Model in Tehran University of Medical Sciences for Research Utilization. Journal of Continuing Education in the Health Professions. 2008; 28(4): 270

معهذا، کاربرد مطلب ذکر شده به صورت ابزار خود ارزیابی حاضر درآمده که طی آن ذینفعان پژوهش در هر سازمان انجام دهنده پژوهش (اعم از دانشگاه، دانشکده، مراکز تحقیقاتی دولتی و خصوصی) می توانند به شناسایی موانع انتقال دانش در سازمان خود بپردازند و بر اساس پاسخ هایی که به دست می آورند، راهکارهای مناسبی بر ای بهبود وضعیت پیشنهاد کنند.

مشابه این ابزار را صندوق حمایت از تحقیقات سیستم های بهداشتی در کانادا Canadian Health System Research Foundation تهیه کرده که برای سازمان های اجرایی استفاده کننده از پژوهش[1] (و نه مشابه ابزار فعلی برای سازمان های تولید کننده دانش) تهیه شده است. تاکید تهیه کنندگان این ابزار بر این است که محتوی این ابزار را می توان بر حسب خصوصیات ویژه یک سازمان تغییر داد. توجه نمائید که روایی و پایایی این ابزار در یک طرح پژوهشی و توسط آزمون و بازآزمون در مراکز تحقیقاتی مورد سنجش قرار گرفته است.

خواهشمند است که پیشنهادات خود را در مورد این ابزار به آدرس الکترونیک kurc@tums.ac.ir و یا rezamajd@tums.ac.ir ارسال نمائید تا نسخه های بعدی این ابزار نیز برای شما ارسال شود.

چگونه از این ابزار استفاده کنیم؟

اعضای شورای پژوهشی و پژوهشگران مرکز تحقیقات یا دانشکده خود را دعوت کنید. بهتر است نمایندگانی از سازمان های استفاده کننده از پژوهش های سازمان خود را نیز دعوت نمایید. این ابزار خودارزیابی را از پیش برای آن ها ارسال کنید تا از قبل گزینه ها را مورد بررسی قرار داده و اگر می خواهند سوالی به آن اضافه کنند.

در بسیاری از قسمت های این متن از استفاده کنندگان پژوهش یا تصمیم گیرندگان نام برده شده است توجه داشته باشید که این گروه ها می توانند سیاست گذاران، مدیران اجرایی، مدیران بیمارستان ها، ارایه کنندگان خدمات بالینی مانند پزشکان، ماماها، پرستاران، داروسازان، دندانپزشکان و ... و نیز گروه های بیماران و یا مردم باشند.

در جلسه ابتدا از همکاران بخواهید که استفاده کنندگان از پژوهش هایی که انجام می دهند را فهرست کنند. سپس، گزینه هایی را که در هر یک از چهار بخش آمده مرور کنید. در هر مورد با یکدیگر بحث کنید و امتیازی را به صورت گروهی به هر یک از گزینه ها بدهید. برخی از سوالات با علامت ستاره (*)مشخص شده اند، اینها سوالاتی هستند که در ارزیابی پایایی این ابزار، ICC کمتر از 70 داشته اند، لذا در مورد این پرسش ها توضیحات لازم را ارائه دهید. قابل ذکر است که بررسی متون و مطالعات انجام شده نشان داده است که این پرسش ها نکات مهمی را در هر سازمان بررسی می نمایند و قرار گرفتن آنها در این ابزار دارای اهمیت می باشد.

---

[1] نسخه فارسی و پیش آزمون شده ابزار سازمان های اجرایی استفاده کننده از پژوهش نیز موجود است که در صورت تمایل می توانید با مکاتبه (به آدرس موجود در متن) نسخه آن را بدست آورد.

نتایجی که به دست می آورید فهرستی از مشکلات سازمان شما در خصوص انتقال دانش است. بهتر است موانعی را که مهمتر هستند انتخاب کنید. برای این کار مشکلات را "اولویت بندی" کنید. سپس برای آن هایی که اولویت بیشتری دارند "مداخله" پیشنهاد کنید.

مراحل کار به صورت زیر خواهد بود:

1- در نظر گرفتن استفاده کنندگان پژوهش هایی که انجام می دهید
2- پاسخ به گزینه های این ابزار خودارزیابی
3- اولویت بندی مسایل شناخته شده
4- انتخاب راه های مداخله برای مسایلی که در اولویت قرار گرفته اند

این ابزار در چهار بخش طراحی شده:

1- *سوال پژوهش: آیا نیازهای تصمیم گیرندگانی که از نتایج تحقیق استفاده می کنند را شناسایی می کنیم و به صورت موضوع پژوهشی درمی آوریم؟*
2- *تولید دانش: آیا شواهدی تولید می کنیم که در تصمیم گیری ها قابل استفاده باشد؟*
3- *انتقال دانش: آیا سازوکارهای مناسب برای انتشار نتایج پژوهش های سازمان به مخاطبین آنها وجود دارد و اقدامات مناسب برای انتقال صورت می گیرد؟*
4- *ترویج استفاده از شواهد:* آیا به تصمیم گیرندگان کمک می کنیم که بتوانند از نتایج پژوهش ها بهتر استفاده کنند؟

در گزینه های این راهنما باید توجه داشت که بسیاری از موضوعاتی که می توانند به تقویت پژوهش می شوند، انتقال دانش را نیز تقویت می کنند. نتیجه اینکه برخی از سوالات به موضوعات عمومی پژوهش از قبیل تقویت شناسایی "سوال پژوهش" و یا "تولید دانش" باز می گردند و تنها قسمت سوم که "*انتقال دانش*" است، تعداد سوال بیشتری دارد. نکته دیگر اینکه این تمام کاری نیست که می توان برای تقویت "ترجمه دانش" کرد، بلکه عمده مداخله هایی است که می توان برای "ترجمه دانش حاصل از پژوهش" در موسساتی که وظیفه آن ها انجام پژوهش است انجام داد. به یاد داشته باشیم که هم راستای این ابزار خودارزیابی، ابزاری دیگر برای سازمان های تصمیم گیرنده وجود دارد.

تعریف برخی واژه ها

- **ترجمه دانش**

تبادل ، سنتز و کاربرد اخلاقی یافته های تحقیق به واسط سامانه پیچیده ای از ارتباطات بین محققین و استفاده کنندگان از دانش به منظور تسریع در جذب منافع دانش از طریق ارتقای سلامت، خدمات، محصولات کارا و نظام مراقبت سلامت.

- **مشارکت**

سه مدل را در خصوص میزان مشارکت تصمیم گیران (سیاست گذاران) می توان توصیف کرد:

1. حمایتگر صرف (formal supporter): در این شرایط تصمیم گیران بطور فعال درگیر فرایند تحقیق نمی باشند: از فعالیتهای جاری تحقیق مطلع نیستند، از اهداف تحقیق حمایت می کنند، شرایط قانونی اجرای تحقیق را فراهم می کنند، دسترسی به منابع را تسهیل می کنند.
2. مستمع پاسخگو (responsive audience): در این شرایط، تصمیم گیر در حدی فعال است که پاسخگوی ایده های محقق بوده و اطلاعات مورد نیاز وی را فراهم کرده و مشاوره های مورد نیاز را ارائه می دهد: اطلاعات مورد نیاز را به محقق میرساند و ارتباطات مورد نیاز را برقرار می کند، ارتباطات ابتدا از جانب محقق بصورت گرفتن مشاوره و برگزاری جلسات تبادل نظر برقرار می شود، محقق از طریق گزارشات مکتوب، تصمیم گیر را در جریان فعالیتهای تحقیق قرار می دهد.
3. شریک اصلی و کامل (integral partner): در این شرایط تصمیم گیر بطور کامل درگیر فرایند تحقیق بوده و شریک موثر در شکل گیری روند تحقیق است: تصمیم گیر به عنوان عضو موثر گروه تحقیق است، ارتباطات ابتدا از جانب تصمیم گیر بصورت برگزاری جلسات با محقق و تبادل نظر برقرار می شود، گزارشات تحقیق پس از هماهنگی و فعالیتهای مشترک بین محقق و سیاست گذار تدوین می شود، سیاست گذار در شکل گیری فرایند تحقیق و نتایج آن موثر است.

- **سازمان تولید کننده پژوهش**

منظور از سازمان تولیدکننده پژوهش، هر مجموعه ای است که توانایی تعریف طرح پژوهشی و تصویب آن را دارد (الزاما تصویب مالی آن منظور نمی باشد و هر مجموعه ای که دارای شورای پژوهشی بوده و می تواند طرح را از نظر علمی تایید نماید مد نظر است) نظیر مراکز تحقیقاتی و دانشکده ها.

در پاسخ به سوالات به چند نکته توجه فرمایید:

- **گزینه ها یک طیف از پاسخ ها را دربر می گیرد مناسب ترین گزینه را انتخاب نمایید.**
- **در صورت وجود هر گونه توضیح، آن را در بخش "توضیحات" مرقوم فرمایید.**
- **در صورتی که سوالی در مورد سازمان شما صدق نمی کند، آن را در بخش "توضیحات" ذکر نمایید.**

بخش نخست: سوال پژوهش

1: آیا می توانیم نیازهای تصمیم گیرندگانی که از نتایج تحقیق استفاده می کنند را شناسایی کنیم و به صورت موضوع پژوهشی درآوریم؟

		وضعیت مناسب است و/یانیاز به مداخله ندارد	4	3	2	وضعیت بسیار نامناسب است و/یا نیاز به مداخله شدید دارد.
1-1	در سازمان ما **فهرست** جامعی از **سازمان** هایی که می توانند از نتایج پژوهشی سازمان ما استفاده کنند وجود دارد.<br>توضیحات:...	5	4	3	2	1
1-2	**بانک های اطلاعاتی** از **مشخصات محققین** و توانمندی های آنها بر حسب واحد های سازمان ما در دسترس سایر سازمان ها می باشد.<br>توضیحات:...	5	4	3	2	1
1-3	**جلسات منظمی** برای تبادل **اولویت های پژوهشی** افراد و یا سازمانهای استفاده کننده از پژوهش، برای شناسایی **اولویت های** آن ها وجود دارد.[1]<br>توضیحات:...	5	4	3	2	1
1-4	**سازمان ما زمینه ها و ظرفیت های پژوهشی خود** را به افراد و یا سازمان های استفاده کننده از این پژوهش ها اعلام کرده است.<br>توضیحات:...	5	4	3	2	1
1-5	برای زمینه سازی اجرای پژوهش های مرتبط، سازمان ما همراه با استفاده کنندگان از نتایج پژوهش (مدیران و سیاست گذاران) جلسات **منظم** و **هدفداری** را برای **توسعه همکاری** و استفاده از **ظرفیت های متقابل** تشکیل می دهد (**شبکه** همکاری).<br>توضیحات:...	5	4	3	2	1

[1] توجه نمایید در این پرسش اولویت های پژوهشی سازمان های استفاده کننده پژوهش مد نظر است.

		وضعیت مناسب است و/یا نیاز به مداخله ندارد	4	3	2	وضعیت بسیار نامناسب است و/یا نیاز به مداخله شدید دارد.
1-6	* در سازمان ما تسهیلاتی (مانند **وب**، **بانک اطلاعاتی و غیره**) برای اطلاع رسانی **اولویت های تحقیقاتی سازمان های دیگر** وجود دارد.<br>توضیحات:..	5	4	3	2	1
1-7	**اولویت‌های پژوهشی سازمان ما** در جلساتی با حضور نمایندگان سازمان‌های اجرایی و یا **استفاده کنندگان از نتایج پژوهش** (نظیر نمایندگان مردم، بیماران و غیره) تعیین می‌گردد[1].<br>توضیحات:..	5	4	3	2	1
1-8	***اولویت های پژوهش سازمان ما** تدوین شده و **فهرست به روز شده** آن در **دسترس** محققین سازمان ما می باشد[2].<br>توضیحات:..	5	4	3	2	1
1-9	***مقدار بودجه** خارج سازمان ، نسبت به بودجه پژوهشی در خود سازمان ، به نحوی است که محققان **ترغیب به استفاده از منابع خارج از سازمان** می شوند.<br>توضیحات:..	5	4	3	2	1
1-10	***فرایند جذب** منابع خارج سازمان ، نسبت به جذب بودجه پژوهشی در خود سازمان، به نحوی است که محققان **ترغیب به استفاده از منابع خارج از** سازمان می شوند (بخش خارج سازمانی فرآیند)<br>توضیحات:..	5	4	3	2	1

[1] توجه نمایید در این پرسش اولویت های پژوهشی سازمان های انجام دهنده پژوهش مد نظر است.

[2] اولویت های پژوهش سازمان انجام دهنده پژوهش ممکن است بر اساس ایده محققین، آینده نگاری، ارزیابی از نیازهای جامعه و نیز نیاز سایر سازمان ها مشخص شده باشد، اما ممکن است با نیازهای پژوهشی سازمان های دیگر (که حتی ممکن است دلیل وجود آن ها یک و یا چند مورد از دلایل ذکر شده باشد) فرق کند.

		وضعیت مناسب است و/یا نیاز به مداخله ندارد	4	3	2	وضعیت بسیار نامناسب است و/یا نیاز به مداخله شدید دارد.
1-11	در صورت جذب **منابع پژوهش خارج از سازمان**، محققین در **زمان کوتاه و به راحتی** می توانند آن را صرف امور پژوهشی نمایند (بخش داخل سازمانی فرآیند)<br>توضیحات:...	5	4	3	2	1
1-12	*در سازمان ما مکانیسم های **تشویقی** برای **جذب اعتبار** پژوهش **خارج از سازمان** وجود دارد.<br>توضیحات:...	5	4	3	2	1
در صورت امکان و در صورتی که تمایل داشته باشد می توانید گزینه های دیگر ی را که برای شرایط سازمان شما مناسب باشد، اضافه نمائید.						
		5	4	3	2	1
		5	4	3	2	1

بخش دوم: تولید دانش

2: آیا شواهدی تولید می کنیم که در تصمیم گیری ها قابل استفاده باشد؟

		وضعیت مناسب است و/یا نیاز به مداخله ندارد	4	3	2	وضعیت بسیار نامناسب است و/یا نیاز به مداخله شدید دارد.
2-1	در مرکز ما پژوهش هایی که منجر به **تولید "پیام قابل انتقال"**[1] با سطح بالایی از شواهد می شوند (مانند انجام مطالعه مروری منظم[2] و یا فعالیت های تولید راهنماهای بالینی و ...) در اولویت های پژوهش قرار داشته و تامین اعتبار می شوند.<br>توضیحات:...	5	4	3	2	1
2-2	گروه هایی که بایستی از نتایج تحقیق استفاده کنند در طراحی تحقیق و یا انجام آن **مشارکت** داده می شوند.<br>توضیحات:...	5	4	3	2	1
2-3	استنباط ما این است که **استفاده کنندگان** از نتایج پژوهش ها به **کیفیت** پژوهش های انجام شده در سازمان ما اطمینان دارند.<br>توضیحات:...	5	4	3	2	1
2-4	در سازمان ما برای انجام هر پژوهش برنامه **تضمین کیفیت** (پروتکل های انجام پرسشگری و یا آموزش کارکنان پژوهش) وجود دارد.<br>توضیحات:...	5	4	3	2	1
2-5	*در سازمان ما در حین انجام پژوهش فعالیت های **کنترل کیفیت** (پایش برنامه اجرا به صورت داخلی توسط گروه تحقیق و یا نظارت خارج از آن) در مورد کلیه طرح ها انجام می گیرد.<br>توضیحات:...	5	4	3	2	1
2-6	زمان بین "مشخص شدن موضوع پژوهش" تا "شروع پژوهش"، قابل قبول است (فرایند **بررسی پیشنهاد پژوهش**).<br>توضیحات:...	5	4	3	2	1

[1] Actionable message
[2] Systematic review

		5	4	3	2	1
		وضعیت مناسب است و/یا نیاز به مداخله ندارد	4	3	2	وضعیت بسیار نامناسب است و /یا نیاز به مداخله شدید دارد.
2-7	*محققین در طراحی پیشنهاد پژوهشی و در انجام طرح های پژوهشی توجه دارند که طرح های کاربردی بایستی در زمان مفید به نتیجه برسند **(مدت زمان انجام طرح ها** و نبود **تاخیر در انجام** آن ها).<br>توضیحات:..	5	4	3	2	1
2-8	زمان بین "پایان پژوهش" تا " ارایه نتایج در قالب گزارش"، قابل قبول است **(فرآیند ارائه نتیجه پژوهش)**.<br>توضیحات:..	5	4	3	2	1
2-9	*در **پیشنهادهای طرح های پژوهشی-پروپوزال** (طرح هایی که استفاده کننده تحقیق آن ارایه دهندگان خدمات، مدیران، سیاست گذاران، گروه های بیماران و یا مردم هستند)، **بودجه برای طرح انتشار نتایج** (غیر از انتشار در مجلات علمی پژوهشی و یا شرکت در کنگره ها) در نظر گرفته می شود.<br>توضیحات:..	5	4	3	2	1
	در صورت امکان و در صورتی که تمایل داشته باشد می توانید گزینه های دیگر ی را که برای شرایط سازمان شما مناسب باشد، اضافه نمائید.					
		5	4	3	2	1
		5	4	3	2	1

بخش سوم: انتقال دانش

3: آیا سازوکارهای مناسب برای انتشار نتایج پژوهش های سازمان به مخاطبین آنها وجود دارد و اقدامات مناسب برای انتقال صورت می گیرد؟

		وضعیت مناسب است و/یا نیاز به مداخله ندارد	4	3	2	وضعیت بسیار نامناسب است و/یا نیاز به مداخله شدید دارد.
3-1	در سازمان ما، شیوه نامه و یا فرآیندی وجود دارد که مشخص می کند که نتایج **کدام یک از پژوهش ها**، باید به گروه های مخاطب (غیر از سایر محققین و سازمان های ارایه دهنده منابع پژوهشی) انتقال یابند.<br>توضیحات:..	5	4	3	2	1
3-2	در سازمان ما نتایج کلیه طرح های پژوهشی پیش از انتقال یا انتشار دانش، مورد **ارزیابی داوران** قرار می گیرد.<br>توضیحات:..	5	4	3	2	1
3-3	*محققین با موضوع "**انتقال دانش حاصل از پژوهش** " و چگونگی انجام آن **آشنایی** دارند.<br>توضیحات:..	5	4	3	2	1
3-4	محققین ما نتیجه تحقیق خود را به صورت "**پیام قابل انتقال**" متناسب با مخاطب آن تبدیل می نمایند.<br>توضیحات:..	5	4	3	2	1
3-5	*محققین ما **مهارت های ارتباطی** را برای انتقال دانش دارند.<br>توضیحات:..	5	4	3	2	1
3-6	*محققین ما می توانند از **خدمات** کسانی استفاده کنند که با **مهارت های انتقال دانش** آشنایی دارند. (وجود افرادی که در سازمان ما با این شرح وظیفه کار می کنند و یا خرید خدمات از افراد و نهادهای خارج از سازمان ما).<br>توضیحات:..	5	4	3	2	1
3-7	*محققین ما **منابع مالی** لازم برای تهیه محتوی مناسب گروه مخاطب نتیجه پژوهش را دارند.<br>توضیحات:..	5	4	3	2	1

		وضعیت مناسب است و/یا نیاز به مداخله ندارد.	4	3	2	وضعیت بسیار نامناسب است و/یا نیاز به مداخله شدید دارد.
3-8	*محققین ما **تجهیزات** لازم برای تهیه محتوی مناسب گروه مخاطب نتیجه پژوهش را دارند.<br>توضیحات:..	5	4	3	2	1
3-9	*محققین ما **زمان** لازم برای تهیه محتوی مناسب گروه مخاطب نتیجه پژوهش را دارند.<br>توضیحات:..	5	4	3	2	1
3-10	***انگیزه لازم برای محققین** سازمان ما (از جمله تشویق، وجود قوانین مناسب در ارتقا و یا ترفیع) برای انتقال دانش وجود دارد.<br>توضیحات:..	5	4	3	2	1
3-11	آموزش انتقال دانش و بهره برداری از نتایج پژوهش ها در **برنامه عمومی آموزش روش تحقیق** وجود دارد.<br>توضیحات:..	5	4	3	2	1
3-12	در هر تحقیق **فهرستی از استفاده کنندگان** از نتایج پژوهش تهیه می شود.<br>توضیحات:..	5	4	3	2	1
3-13	***ساختار** (مانند دفتر و یا واحد سازمانی) و یا **نیروی انسانی** لازم برای تقویت انتقال دانش، با توجه به مقدار تولید پژوهش های قابل انتقال به تصمیم گیرندگان، در سازمان ما وجود دارد.<br>توضیحات:..	5	4	3	2	1
3-14	*مدیران پژوهشی سازمان ما **نیازهای** گروه های مختلف محققین را (به تفکیک رشته تحصیلی – گروه و یا غیره) در زمینه انتقال دانش ارزیابی کرده و برنامه مداخله مناسب برای آنها اجرا می کنند.<br>توضیحات:..	5	4	3	2	1

		وضعیت مناسب است و/یا نیاز به مداخله ندارد.	4	3	2	وضعیت بسیار نامناسب است و/یا نیاز به مداخله شدید دارد.
3-15	چارچوب (فرمت) **مجلات علمی** که مقالات حاصل از پژوهش ها را منتشر می کنند به شکلی است که تصمیم گیرندگان می توانند در صورت نیاز از **"پیام قابل انتقال"** تحقیق به راحتی مطلع شوند.<br>توضیحات:...	5	4	3	2	1
3-16	***زمان ارسال مقاله تا انتشار** آن در مجلات به نحوی است که مداخله های حاصل از پژوهش در زمان معقول به نتیجه برسند (با توجه به نیاز به دسترسی سریع تصمیم گیران به نتایج تحقیق)[1]<br>توضیحات:...	5	4	3	2	1
3-17	**چارچوب گزارش نهایی** طرح های پژوهشی به صورتی است که به سادگی سازمان ها و یا افراد استفاده کننده از تحقیق می توانند پیام قابل انتقال را شناسایی کنند.<br>توضیحات:...	5	4	3	2	1
3-18	نتایج پژوهش از طریق **وب** و یا **بانک الکترونیکی** توسط سازمان ما منتشر می شود.<br>توضیحات:...	5	4	3	2	1
3-19	**جلسات ارایه نتایج پژوهش ها** به تصمیم گیرندگان برگزار می شود.<br>توضیحات:...	5	4	3	2	1
3-20	*سازمان ما **برنامه منظمی با رسانه های عمومی و اختصاصی** و گروه های مخاطب ما (مانند نشریات مربوط به زنان و یا جوانان) برای انتقال دانش حاصل از پژوهش دارد.<br>توضیحات:...	5	4	3	2	1
3-21	***قوانین** حمایت از مالکیت معنوی محققینی که قبل از انتشار مطلب در مجلات به انتشار نتایج پژوهش اقدام می کنند، وجود دارد.<br>توضیحات:...	5	4	3	2	1

1- نگارندگان کاملا توجه دارند که ممکن است مجلاتی که معمولا مقالات همکاران را منتشر می کنند، خارج از سازمان باشند ولی شاید بتوان مداخله هایی در این زمینه انجام داد، مانند تصمیم به انتشارمجله، ترغیب و حمایت از انتشار در مجلاتی که به صورت الکترونیک انتشار می یابند. برای همین این سوالات را برای بحث باقی گذاشته اند.

		5	4	3	2	1
		وضعیت مناسب است و/یا نیاز به مداخله ندارد	4	3	2	وضعیت بسیار نامناسب است و/یا نیاز به مداخله شدید دارد.
3-22	**میزان استفاده از شواهد** (پژوهش های داخلی و یا خارجی) **توسط تصمیم گیرندگان**، از موضوعات تحقیق در سازمان ما می باشد.<br>توضیحات:..	5	4	3	2	1
3-23	*محققین ما **میزان استفاده از نتایج پژوهش** های سازمان ما توسط تصمیم گیرندگان را مورد بررسی قرار می دهند (در قالب بخشی از طرح تحقیق و یا پیشنهاد طرح پژوهشی مجزا).<br>توضیحات:..	5	4	3	2	1
3-24	*محققین سازمان ما **موانع** احتمالی **تغییر رفتار تصمیم گیرندگان** برای استفاده از نتیجه پژوهش های آن ها را شناسایی می کنند.<br>توضیحات:..	5	4	3	2	1
3-25	**ملاک هایی برای ارزیابی** فعالیت های محققین برای انتقال دانش حاصل از پژوهش در سازمان ما وجود دارد.<br>توضیحات:..	5	4	3	2	1
	در صورت امکان و در صورتی که تمایل داشته باشد می توانید گزینه های دیگر ی را که برای شرایط سازمان شما مناسب باشد، اضافه نمائید.					
		5	4	3	2	1
		5	4	3	2	1

بخش چهارم: ترویج استفاده از شواهد

4: آیا به تصمیم گیرندگان کمک می کنیم که بتوانند از نتایج پژوهش ها بهتر استفاده کنند؟

		5 وضعیت مناسب است و/یا نیاز به مداخله ندار د	4	3	2	1 وضعیت بسیار نامناسب است و/یا نیاز به مداخله شدید دارد.
4-1	*برنامه های آموزشی مانند "**پزشکی مبتنی بر شواهد**" و یا "**تصمیم گیری مبتنی بر شواهد**" برای ارایه دهندگان خدمات و یا مدیران برگزار می کنیم.<br>توضیحات:...	5	4	3	2	1
4-2	برنامه هایی برای ترویج استفاده تصمیم گیران از **ابزارهایی** که تصمیم گیری مبتنی بر شواهد را تقویت می کند، در دسترس بوده و اجرا می شود (مانند تولید شواهد "مطالعات مروری سیستماتیک و راهنماهای بالینی").<br>توضیحات:...	5	4	3	2	1
4-3	*محققین ما در کمیته های فنی کمک به تصمیم گیری (تصمیم گیری سازمان های اجرایی، مدیریت بیمارستان و نیز گروه های حمایت کننده از سلامت بیماران و مردم) نقش فعالی را ایفا می کنند.<br>توضیحات:...	5	4	3	2	1
4-4	*برای تصمیم گیرندگان پیام هایی برای پی گیری نتایج پژوهش هایی که قبلا نتایج آن را فرستاده بودیم ("**یادآور**" Reminder) می فرستیم.<br>توضیحات:...	5	4	3	2	1
در صورت امکان و در صورتی که تمایل داشته باشد می توانید گزینه های دیگر ی را که برای شرایط سازمان شما مناسب باشد، اضافه نمائید.						
		5	4	3	2	1
		5	4	3	2	1

تهران، بلوار کشاورز، خیابان 16 آذر، خیابان نصرت، پلاک 12
تلفن/فاکس: 02166495859 – 02166419763- 02166952530
ایمیل: kurc@tums.ac.ir
